# Structural characterization of two benzene-1,2-di­amine complexes of zinc chloride: a mol­ecular compound and a co-crystal salt

**DOI:** 10.1107/S2056989016010033

**Published:** 2016-06-24

**Authors:** Patricia L. Zick, David K. Geiger

**Affiliations:** a Department of Chemistry, SUNY-College at Geneseo, Geneseo, NY 14454, USA

**Keywords:** crystal structure, benzene-1,2-di­amine, zinc chloride, co-crystal salt

## Abstract

The structures of two zinc complexes containing bidentate benzene-1,2-di­amine ligands are reported. (Benzene-1,2-di­amine-κ^2^ *N*,*N*′)di­chloro­idozinc, [ZnCl_2_(C_6_H_8_N_2_)], (I), displays a distorted tetra­hedral coordination sphere for the metal cation. The di­amine ligand and the Zn atom reside on a crystallographic mirror plane. In the 1:1 co-crystal salt *trans*-di­aqua­bis­(4,5-di­methyl­benzene-1,2-di­amine-κ^2^ *N*,*N*′)zinc chloride–4,5-di­methyl­benzene-1,2-di­amine (1/1), [Zn(C_8_H_12_N_2_)_2_(H_2_O)_2_]Cl_2_·2C_8_H_12_N_2_, (II), the zinc(II) complex cation exhibits a tetra­gonally distorted octa­hedral coordination sphere. The Zn atom sits on a crystallographically imposed inversion center and the di­amine ligands are tilted 30.63 (6)° with respect to the ZnN_4_ plane. Both complexes exhibit extensive hydrogen bonding. In (I), a stacked-sheet extended structure parallel to (101) is observed. In (II), the co-crystallized di­amine is hydrogen-bonded to the complex cation *via* O—H⋯N and N—H⋯N linkages. These units are in turn linked into planes along (200) by O—H⋯Cl and N—H⋯Cl hydrogen bonds.

## Abstract 2

The structures of two zinc complexes containing bidentate benzene-1,2-di­amine ligands are reported. (Benzene-1,2-di­amine-κ^2^ *N*,*N*′)di­chloro­idozinc displays a distorted tetra­hedral geometry. The 1:1 co-crystal salt *trans*-di­aqua­bis­(4,5-di­methyl­benzene-1,2-di­amine- κ^2^ *N*,*N*′)zinc chloride 4,5-di­methyl­benzene-1,2-di­amine exhibits a tetra­gonally distorted octa­hedral zinc coordination sphere.

## Chemical context   

Zinc complexes bearing aryl di­imine and/or heterocyclic ligands have been shown to emit brightly in the blue region of the spectrum (DeStefano & Geiger, 2016 ▸; Tan *et al.*, 2012 ▸; Liu *et al.*, 2010 ▸; Xu *et al.*, 2008 ▸; Yue *et al.*, 2006 ▸; Singh *et al.*, 2011 ▸; Wang *et al.*, 2010 ▸). These complexes have potential use in photooptical devices because of their high thermal stability and the ability to tune their color by varying ancillary ligands and coordination geometry (Xu *et al.*, 2008 ▸). Most of the compounds explored have acetate ligands. Substituting acetate with halide ligands provides an avenue for modulating the electronic structure of the complex and, hence, the carrier transport character. Toward that end, we have characterized several zinc complexes possessing benzene-1,2-di­amine ligands (Geiger, 2012 ▸; Geiger & Parsons, 2014 ▸) and substituted benzimidazole ligands (DeStefano & Geiger, 2016 ▸). The benzene-1,2-di­amine-containing complexes previously reported have a monodentate di­amine coordination mode. We report herein two new zinc complexes containing bidentate benzene-1,2-di­amine ligands: (benzene-1,2-di­amine-κ^2^ *N*,*N*′)di­chlor­idozinc, (I), and the 1:1 co-crystal salt *trans*-di­aqua­bis­(4,5-di­methyl­benzene-1,2-di­amine-κ^2^ *N,N′*)zinc chloride 4,5-di­methyl­benzene-1,2-di­amine, (II).
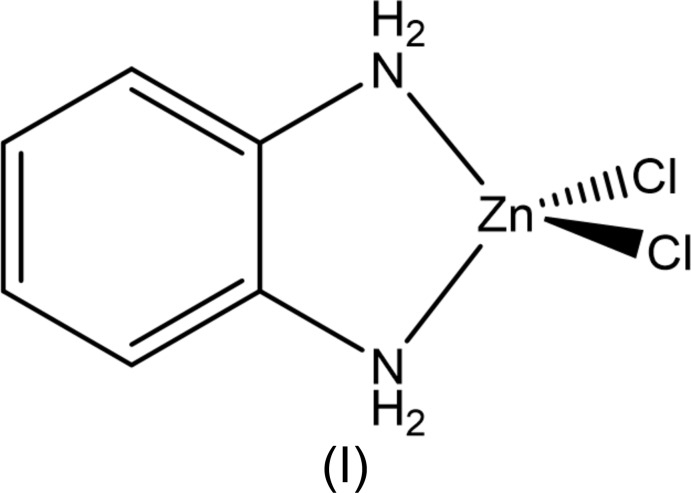
 
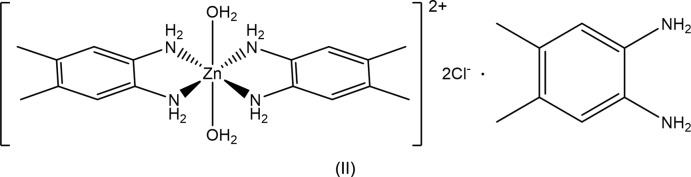


## Structural commentary   

**Figure 1:**
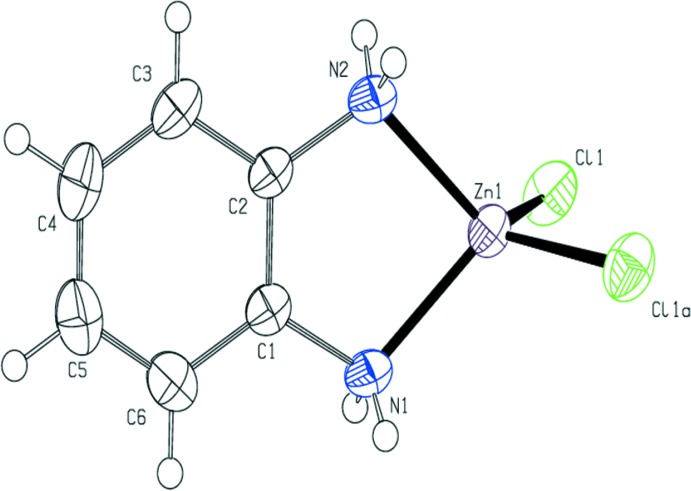
The mol­ecular structure of (I*a*), showing the atom-labeling scheme. Anisotropic displacement parameters are drawn at the 50% probability level. [Symmetry code: (*a*) *x*, −*y* + 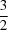, *z*.]

As seen in Fig. 1 ▸, compound (I) exhibits a distorted tetra­hedral coordination sphere for the metal cation. Tables 1 ▸ and 2 ▸ give relevant geometric parameters found in the coordination sphere. The di­amine ligand and the Zn atom sit on a mirror plane and, hence, are rigorously planar as a result of the symmetry constraint. The Zn—N bond lengths observed at the two temperatures are the same within the calculated s.u.s. The Zn—Cl bond lengths differ within the s.u.s, with the 200 K structure being 0.0030 (5) Å longer. The bond lengths observed at both temperatures fall within the s.u. of the average value [2.221 (19) Å] of similar complexes but the Cl—Zn—Cl bond angles are smaller than the average of the values [115 (1)°] reported for similar Zn^II^ dichlorides in a tetra­hedral environment (Shi *et al.*, 2010 ▸; You, 2005 ▸; Lee *et al.*, 2007 ▸).

**Figure 2:**
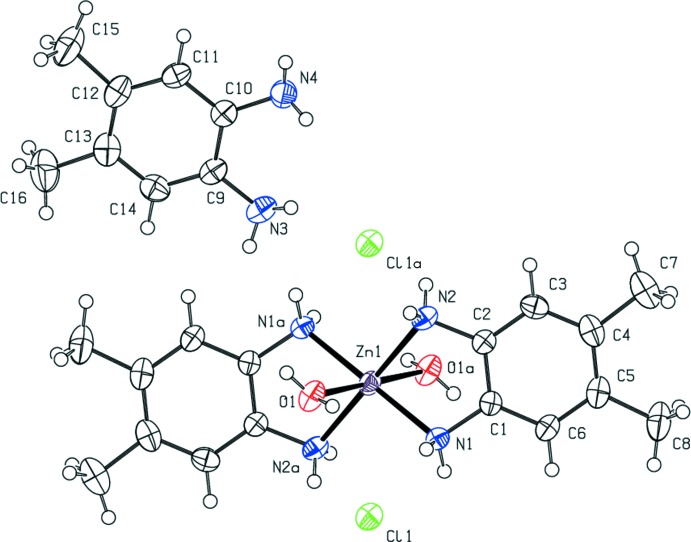
The mol­ecular structure of (II) showing the atom-labeling scheme. Anisotropic displacement parameters are drawn at the 50% probability level. [Symmetry code: (*a*) −*x* + 1, −*y* + 1, −*z* + 1.]

Compound (II) consists of a Zn^II^ complex cation with two bidentate 4,5-di­methyl­benzene-1,2-di­amine ligands and *trans* water ligands, chloride counter-ions and a non-coordinating mol­ecule of 4,5-di­methyl­benzene-1,2-di­amine. The compound is thus classified as a co-crystal salt (Grothe *et al.*, 2016 ▸). A representation of (II) is found in Fig. 2 ▸. The Zn^II^ ion sits on a crystallographically imposed center of symmetry and has a tetra­gonally distorted octa­hedral coordination geometry. The observed Zn—O bond length (Table 3 ▸) is significantly longer than the average of the values [2.14 (3) Å] reported for similar *trans* aqua zinc(II) complexes (Necefoglu *et al.*, 2001 ▸; İbrahim *et al.*, 2006 ▸; Karimnejad *et al.*, 2011 ▸; Gallardo *et al.*, 2008 ▸; Li *et al.*, 2012 ▸) and the range [2.008 (3) to 2.147 (3) Å] found in the hexa­aqua­zinc(II) cation (Lian *et al.*, 2009 ▸). However, it is close to the 2.2057 (16) Å found in the similar cation of *trans*-di­aqua­bis­(cyclo­hexane-1,2-di­amine)­zinc dichloride (Karimnejad *et al.*, 2011 ▸). The plane of the 4,5-di­methyl­benzene-1,2-di­amine ligand is canted 30.63 (6) Å out of the ZnN_4_ coordin­ation plane. The nitro­gen atoms of the di­amine ligand are 0.022 (3) and 0.131 (3) Å out of the benzene plane for N1 and N2, respectively. For the co-crystallized di­amine, N3 and N4 are 0.139 (3) and 0.088 (3) Å out of the plane, respectively.

## Supra­molecular features   

**Figure 3:**
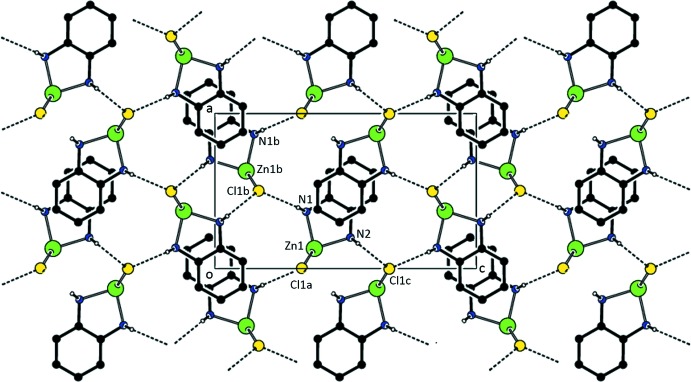
A view of the parallel sheets found in (I). Only H atoms involved in the N—H⋯Cl inter­actions are shown. [Symmetry codes: (*a*) *x*, −*y* + 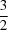, *z*; (*b*) *x* + 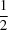, −*y* + 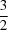, −*z* + 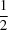; (*c*) −*x*, −*y* + 1, −*z* + 1.]

As seen in Figs. 3 ▸ and 4 ▸ and Tables 4 ▸ and 5 ▸, N1—H1⋯Cl hydrogen bonds between adjacent mol­ecules result in strips of mol­ecules of (I) along [100]. The strips form planes parallel to (101). Additional N2—H2⋯Cl bonds join the strips to form the three-dimensional network.

**Figure 5:**
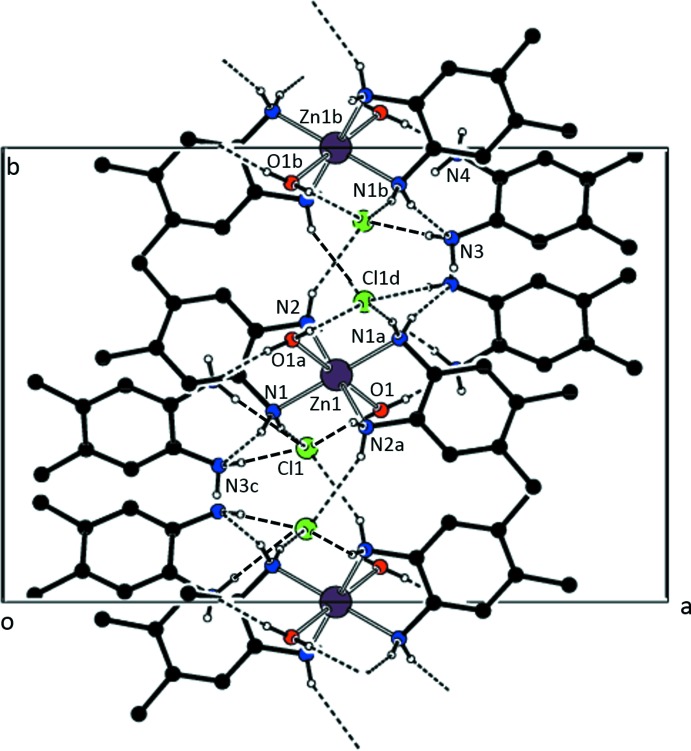
A view of the hydrogen-bonded network of (II) resulting in slabs along (200). Only H atoms bonded to the nitro­gen atoms are shown. [Symmetry codes: (*a*) −*x* + 1, −*y* + 1, −*z* + 1; (*b*) −*x* + 1, *y* + 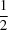, −*z* + 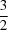; (*c*) −*x* + 1, *y* − 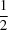, −*z* + 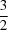; (*d*) −*x* + 1, −*y* + 1, −*z* + 2.]

Fig. 5 ▸ presents a view of the hydrogen-bonding network in (II). N—H⋯N and O—H⋯N hydrogen bonds connect inversion-related co-crystallized 4,5-di­methyl­benzene-1,2-di­amine mol­ecules to the complex cation (see Table 6 ▸). Additional N—H⋯Cl and O—H⋯Cl hydrogen bonds join the units, forming planes parallel to (200).

## Database survey   

The structures of the tetra­hedral complexes bis­(acetato-*κO*)(benzene-1,2-di­amine-*κN*)zinc (Mei *et al.*, 2009 ▸) and bis(acetato-*κO*)(4,5-di­methyl­benzene-1,2-di­amine-*κN*)zinc (Geiger, 2012 ▸) have been reported. Poly[[tris­(*μ*_2_-acetato-κ^2^ *O*:*O′*)(4-chloro­benzene-1,2-di­amine-*κN*)(*μ*_3_-hydroxido)dizinc] ethanol monosolvate] exhibits alternating octa­hedral and tetra­hedral zinc coordination modes (Geiger & Parsons, 2014 ▸). Di­chlorido­[*N*-(2-pyridyl­methyl­idene)benzene-1,4-di­amine]­zinc has a tetra­hedral coordination sphere with inter­molecular N—H⋯Cl hydrogen bonds (Shi *et al.*, 2010 ▸). Di­chlorido­[*N,N,N′,N′*-tetra­methyl­cyclo­hexane-1,2-di­amine-κ^2^ *N*,*N*′]zinc displays a tetra­hedral coordination geometry (Lee *et al.*, 2007 ▸). For examples of zinc complexes with the metal in octahedral coordination including *trans* water ligands, see İbrahim *et al.* (2006 ▸); Necefoglu *et al.* (2001 ▸); Karimnejad *et al.* (2011 ▸). A tetra­gonally distorted octa­hedral zinc complex that contains both a mono- and a bidentate benzene-1,2-di­amine ligand (Qian *et al.*, 2007 ▸) and a distorted octa­hedral complex with *trans* monodentate benzene-1,2-di­amine ligands (Ovalle-Marroquín *et al.*, 2002 ▸) have been reported.

## Synthesis and crystallization   

Compound (I) was prepared by mixing a solution of 100. mg (0.734 mmol) zinc chloride dissolved in approximately 5 mL ethanol with a solution of 238 mg (2.20 mmol) benzene-1,2-di­amine dissolved in approximately 5 mL ethanol. The mixture became cloudy with a fine white precipitate. After the addition of 4 drops of 6 *M* HCl, the mixture was gently heated, filtered and allowed to slowly evaporate. After two days, 0.0273 g (0.117 mmol, 15% yield) of clear, colorless crystals were isolated, which were used for data collection. The diffraction pattern showed signs of degradation as the temperature was lowered to 200 K from 300 K and so data sets were collected at both temperatures.

Compound (II) was prepared by combining solutions of 100 mg (0.734 mmole) zinc chloride in a few mL of ethanol and 300 mg (2.20 mmol) 4,5-di­methyl­benzene-1,2-di­amine in a few mL of ethanol. After the addition of 4 drops of 6 *M* HCl, the mixture was gently heated and filtered. The filtrate was divided into three portions and each allowed to slowly evaporate. After several days, a small number of clear, colorless crystals in the shape of hexa­gonal plates were isolated, one of which was used for data collection.

## Refinement details   

Crystal data, data collection and structure refinement details are summarized in Table 7 ▸. For compound (I), data sets were collected at 300 K (I*a*) and 200 K (I*b*). The diffraction pattern showed clear degradation at the lower temperature. Examination of the crystal subjected to the cold stream showed fractures that were not previously present. As seen in Table 7 ▸, the cell constant s.u.s, *R* values and *S* values are lower for the 300 K data set.

For both (I) and (II), all hydrogen atoms were located in difference Fourier maps. For (I), all hydrogen atoms bonded to the nitro­gen atoms were refined freely, including isotropic displacement parameters. For (I*a*), the hydrogen atoms bonded to the benzene carbon atoms were refined using a riding model with C—H = 0.93 Å and *U*_iso_(H) = 1.2*U*_eq_(C), whereas these hydrogen atoms were refined with C—H = 0.95 Å and *U*_iso_(H) = 1.2*U*_eq_(C) for (I*b*).

For (II), the amine hydrogen atoms of the non-coordinating 4,5-di­methyl­benzene-1,2-di­amine were refined freely, including the isotropic displacement parameters. For the hydrogen atoms of the coordinating amines, the atomic coord­inates were refined freely with *U*_iso_(H) = 1.2*U*_eq_(N). The hydrogen atoms of the water ligands were refined freely, including the isotropic displacement parameters. The methyl hydrogen atoms were refined with C—H = 0.98 Å and *U*_iso_(H) = 1.5*U*_eq_(C).

## Supplementary Material

Crystal structure: contains datablock(s) global, Ia, II, Ib. DOI: 10.1107/S2056989016010033/lh5817sup1.cif

Structure factors: contains datablock(s) Ia. DOI: 10.1107/S2056989016010033/lh5817Iasup2.hkl

Supporting information file. DOI: 10.1107/S2056989016010033/lh5817Iasup5.mol

Structure factors: contains datablock(s) II. DOI: 10.1107/S2056989016010033/lh5817IIsup3.hkl

Supporting information file. DOI: 10.1107/S2056989016010033/lh5817IIsup7.mol

Structure factors: contains datablock(s) Ib. DOI: 10.1107/S2056989016010033/lh5817Ibsup4.hkl

Supporting information file. DOI: 10.1107/S2056989016010033/lh5817Ibsup6.mol

CCDC references: 1486732, 1486731, 1486730

Additional supporting information:  crystallographic information; 3D view; checkCIF report

## Figures and Tables

**Figure 4:**
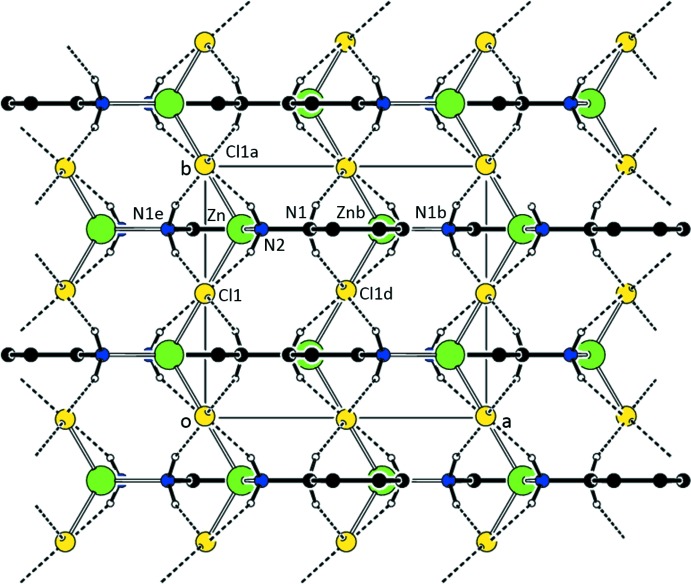
A view of the parallel sheets found in (I). Only H atoms involved in the N—H⋯Cl inter­actions are shown. [Symmetry codes: (*a*) *x*, −*y* + 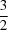, *z*; (*b*) *x* + 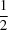, −*y* + 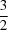, −*z* + 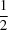; (*d*) *x* + 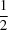, *y*, −*z* + 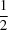; (*e*) *x* − 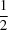, −*y* + 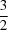, −*z* + 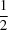.]

**Table 1:** Selected geometric parameters (Å, °) for (I*a*)

Zn1—Cl1	2.2271 (5)	Zn1—N2	2.0454 (18)
Zn1—N1	2.0449 (19)		
			
Cl1—Zn1—Cl1^i^	110.82 (2)	N1—Zn1—Cl1	113.82 (3)
N1—Zn1—N2	85.53 (8)	N2—Zn1—Cl1	115.42 (3)

Symmetry code: (i) 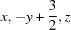.

**Table 2:** Selected geometric parameters (Å, °) for (I*b*)

Zn1—Cl1	2.2301 (5)	Zn1—N2	2.045 (3)
Zn1—N1	2.047 (2)		
			
Cl1—Zn1—Cl1^i^	110.70 (3)	N1—Zn1—Cl1	113.89 (4)
N1—Zn1—N2	85.45 (10)	N2—Zn1—Cl1	115.46 (3)

Symmetry code: (i) 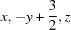.

**Table 3:** Selected geometric parameters (Å, °) for (II)

Zn1—N1	2.1214 (15)	Zn1—O1	2.2410 (15)
Zn1—N2	2.1442 (17)		
			
N1^i^—Zn1—N2	100.31 (6)	N1—Zn1—O1	92.18 (7)
N1—Zn1—N2	79.69 (6)	N2—Zn1—O1	93.22 (7)

Symmetry code: (i) 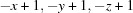.

**Table 4:** Hydrogen-bond geometry (Å, °) for (I*a*)

*D*—H⋯*A*	*D*—H	H⋯*A*	*D*⋯*A*	*D*—H⋯*A*
N1—H1⋯Cl1^ii^	0.86 (2)	2.59 (2)	3.3618 (16)	150.7 (18)
N2—H2⋯Cl1^iii^	0.85 (2)	2.52 (2)	3.3204 (16)	157 (2)

Symmetry codes: (ii) 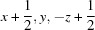; (iii) 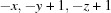.

**Table 5:** Hydrogen-bond geometry (Å, °) for (I*b*)

*D*—H⋯*A*	*D*—H	H⋯*A*	*D*⋯*A*	*D*—H⋯*A*
N1—H1⋯Cl1^ii^	0.83 (3)	2.61 (3)	3.368 (2)	152 (2)
N2—H2⋯Cl1^iii^	0.83 (3)	2.53 (3)	3.327 (2)	160 (2)

Symmetry codes: (ii) 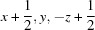; (iii) 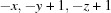.

**Table 6:** Hydrogen-bond geometry (Å, °) for (II)

*D*—H⋯*A*	*D*—H	H⋯*A*	*D*⋯*A*	*D*—H⋯*A*
O1—H1*WA*⋯N4^ii^	0.81 (3)	2.13 (3)	2.924 (3)	164 (2)
O1—H1*WB*⋯Cl1^iii^	0.80 (3)	2.31 (3)	3.1083 (17)	173 (2)
N1—H1*A*⋯Cl1^iii^	0.84 (2)	2.55 (2)	3.3551 (18)	160.3 (18)
N1—H1*B*⋯N3^iv^	0.85 (2)	2.31 (2)	3.137 (3)	162.6 (18)
N2—H2*A*⋯Cl1^v^	0.87 (2)	2.63 (2)	3.4401 (19)	155.3 (18)
N2—H2*B*⋯Cl1^i^	0.81 (2)	2.57 (2)	3.3105 (18)	154 (2)
N3—H3*A*⋯Cl1^v^	0.84 (3)	2.68 (3)	3.516 (2)	174 (2)
N3—H3*B*⋯Cl1^vi^	0.87 (3)	2.89 (2)	3.3284 (19)	112.9 (18)
N4—H4*B*⋯Cl1^v^	0.87 (3)	2.50 (3)	3.355 (2)	171 (2)

Symmetry codes: (i) 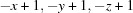; (ii) 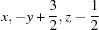; (iii) 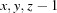; (iv) 
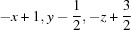
; (v) 
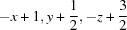
; (vi) 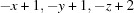.

**Table 7:** Experimental details

	(I*a*)	(I*b*)	(II)
Crystal data			
Chemical formula	[ZnCl_2_(C_6_H_8_N_2_)]	[ZnCl_2_(C_6_H_8_N_2_)]	[Zn(C_8_H_12_N_2_)_2_(H_2_O)_2_]Cl_2_·2C_8_H_12_N_2_
*M*_r_	244.41	244.41	717.08
Crystal system, space group	Orthorhombic, *P* *n* *m* *a*	Orthorhombic, *P* *n* *m* *a*	Monoclinic, *P*2_1_/*c*
Temperature (K)	300	200	200
*a*, *b*, *c* (Å)	8.4039 (9), 7.5206 (7), 14.1667 (15)	8.4152 (12), 7.5141 (9), 14.199 (2)	18.529 (2), 12.6227 (16), 7.8691 (8)
α, β, γ (°)	90, 90, 90	90, 90, 90	90, 94.665 (4), 90
*V* (Å^3^)	895.37 (16)	897.8 (2)	1834.4 (4)
*Z*	4	4	2
Radiation type	Mo *K*α	Mo *K*α	Mo *K*α
μ (mm^−1^)	3.27	3.27	0.86
Crystal size (mm)	0.60 × 0.30 × 0.20	0.60 × 0.30 × 0.20	0.60 × 0.40 × 0.10

Data collection			
Diffractometer	Bruker SMART X2S benchtop	Bruker SMART X2S benchtop	Bruker SMART X2S benchtop
Absorption correction	Multi-scan (*SADABS*; Bruker, 2013 ▸)	Multi-scan (*SADABS*; Bruker, 2013 ▸)	Multi-scan (*SADABS*; Bruker, 2013 ▸)
*T*_min_, *T*_max_	0.39, 0.56	0.40, 0.56	0.66, 0.92
No. of measured, independent and observed [*I* > 2σ(*I*)] reflections	9435, 1129, 1026	4392, 1090, 992	25430, 3619, 2920
*R*_int_	0.039	0.040	0.060
(sin θ/λ)_max_ (Å^−1^)	0.658	0.649	0.617

Refinement			
*R*[*F* ^2^ > 2σ(*F* ^2^)], *wR*(*F* ^2^), *S*	0.020, 0.051, 1.09	0.027, 0.074, 1.16	0.031, 0.079, 1.05
No. of reflections	1129	1090	3619
No. of parameters	72	72	245
H-atom treatment	H atoms treated by a mixture of independent and constrained refinement	H atoms treated by a mixture of independent and constrained refinement	H atoms treated by a mixture of independent and constrained refinement
Δρ_max_, Δρ_min_ (e Å^−3^)	0.26, −0.40	0.41, −0.58	0.31, −0.22

Computer programs: *APEX2* and *SAINT* (Bruker, 2013 ▸), *SHELXS97* (Sheldrick, 2008 ▸), *SHELXL2014* (Sheldrick, 2015 ▸), *PLATON* (Spek, 2009 ▸), *Mercury* (Macrae *et al.*, 2006 ▸) and *publCIF* (Westrip, 2010 ▸).

## supplementary crystallographic information

### (Ia) (Benzene-1,2-diamine-κ2N,N')dichloroidozinc Crystal data

**Table:**

[ZnCl_2_(C_6_H_8_N_2_)]	*D*_x_ = 1.813 Mg m^−^^3^
*M**_r_* = 244.41	Mo *K*α radiation, λ = 0.71073 Å
Orthorhombic, *P**n**m**a*	Cell parameters from 6300 reflections
*a* = 8.4039 (9) Å	θ = 2.8–29.2°
*b* = 7.5206 (7) Å	µ = 3.27 mm^−^^1^
*c* = 14.1667 (15) Å	*T* = 300 K
*V* = 895.37 (16) Å^3^	Parallelpiped, colorless
*Z* = 4	0.60 × 0.30 × 0.20 mm
*F*(000) = 488	

### (Ia) (Benzene-1,2-diamine-κ2N,N')dichloroidozinc Data collection

**Table:**

Bruker SMART X2S benchtop diffractometer	1129 independent reflections
Radiation source: sealed microfocus tube	1026 reflections with *I* > 2σ(*I*)
Doubly curved silicon crystal monochromator	*R*_int_ = 0.039
Detector resolution: 8.3330 pixels mm^-1^	θ_max_ = 27.9°, θ_min_ = 2.8°
/w scans	*h* = −11→11
Absorption correction: multi-scan (SADABS; Bruker, 2013)	*k* = −9→8
*T*_min_ = 0.39, *T*_max_ = 0.56	*l* = −18→15
9435 measured reflections	

### (Ia) (Benzene-1,2-diamine-κ2N,N')dichloroidozinc Refinement

**Table:**

Refinement on *F*^2^	Primary atom site location: structure-invariant direct methods
Least-squares matrix: full	Secondary atom site location: difference Fourier map
*R*[*F*^2^ > 2σ(*F*^2^)] = 0.020	Hydrogen site location: mixed
*wR*(*F*^2^) = 0.051	H atoms treated by a mixture of independent and constrained refinement
*S* = 1.09	*w* = 1/[σ^2^(*F*_o_^2^) + (0.0226*P*)^2^ + 0.2253*P*] where *P* = (*F*_o_^2^ + 2*F*_c_^2^)/3
1129 reflections	(Δ/σ)_max_ = 0.001
72 parameters	Δρ_max_ = 0.26 e Å^−^^3^
0 restraints	Δρ_min_ = −0.40 e Å^−^^3^

### (Ia) (Benzene-1,2-diamine-κ2N,N')dichloroidozinc Special details

**Table:**

Geometry. All esds (except the esd in the dihedral angle between two l.s. planes) are estimated using the full covariance matrix. The cell esds are taken into account individually in the estimation of esds in distances, angles and torsion angles; correlations between esds in cell parameters are only used when they are defined by crystal symmetry. An approximate (isotropic) treatment of cell esds is used for estimating esds involving l.s. planes.
Refinement. Refined as a 2-component inversion twin.

### (Ia) (Benzene-1,2-diamine-κ2N,N')dichloroidozinc Fractional atomic coordinates and isotropic or equivalent isotropic displacement parameters (Å^2^)

**Table:**

	*x*	*y*	*z*	*U*_iso_*/*U*_eq_	
Zn1	0.12910 (3)	0.75	0.38035 (2)	0.03301 (10)	
N1	0.3667 (2)	0.75	0.34928 (13)	0.0359 (5)	
H1	0.393 (2)	0.656 (3)	0.3182 (15)	0.050 (6)*	
N2	0.1999 (2)	0.75	0.51849 (13)	0.0362 (5)	
H2	0.162 (3)	0.660 (3)	0.5468 (16)	0.057 (6)*	
Cl1	0.00181 (5)	0.50621 (6)	0.33277 (3)	0.04250 (13)	
C1	0.4560 (3)	0.75	0.43712 (14)	0.0301 (4)	
C2	0.3724 (2)	0.75	0.52146 (14)	0.0293 (4)	
C3	0.4541 (3)	0.75	0.60645 (15)	0.0406 (5)	
H3	0.3982	0.75	0.6631	0.049*	
C4	0.6191 (3)	0.75	0.60699 (19)	0.0496 (7)	
H4	0.6742	0.75	0.6639	0.059*	
C5	0.7007 (3)	0.75	0.5231 (2)	0.0508 (7)	
H5	0.8114	0.75	0.5236	0.061*	
C6	0.6208 (3)	0.75	0.43830 (19)	0.0434 (6)	
H6	0.6774	0.75	0.3819	0.052*	

### (Ia) (Benzene-1,2-diamine-κ2N,N')dichloroidozinc Atomic displacement parameters (Å^2^)

**Table:**

	*U*^11^	*U*^22^	*U*^33^	*U*^12^	*U*^13^	*U*^23^
Zn1	0.03143 (15)	0.04349 (19)	0.02409 (14)	0	−0.00277 (9)	0
N1	0.0353 (10)	0.0523 (14)	0.0200 (8)	0	0.0028 (7)	0
N2	0.0357 (10)	0.0506 (14)	0.0223 (8)	0	0.0037 (7)	0
Cl1	0.0528 (3)	0.0391 (3)	0.0355 (2)	−0.00706 (18)	−0.00622 (16)	0.00356 (16)
C1	0.0339 (11)	0.0307 (12)	0.0258 (9)	0	0.0001 (8)	0
C2	0.0342 (11)	0.0308 (12)	0.0228 (9)	0	−0.0010 (7)	0
C3	0.0499 (14)	0.0474 (15)	0.0244 (10)	0	−0.0057 (9)	0
C4	0.0485 (15)	0.0559 (18)	0.0443 (14)	0	−0.0201 (11)	0
C5	0.0354 (13)	0.0582 (18)	0.0587 (15)	0	−0.0093 (11)	0
C6	0.0332 (12)	0.0541 (17)	0.0429 (13)	0	0.0041 (9)	0

### (Ia) (Benzene-1,2-diamine-κ2N,N')dichloroidozinc Geometric parameters (Å, º)

**Table:**

Zn1—Cl1^i^	2.2271 (5)	C5—C6	1.377 (4)
Zn1—Cl1	2.2271 (5)	C5—H5	0.93
Zn1—N1	2.0449 (19)	C4—C5	1.372 (4)
Zn1—N2	2.0454 (18)	C4—H4	0.93
N2—C2	1.451 (3)	C3—C4	1.387 (4)
N2—H2	0.85 (2)	C3—H3	0.93
N1—C1	1.453 (3)	C2—C3	1.386 (3)
N1—H1	0.86 (2)	C1—C2	1.386 (3)
C6—H6	0.93	C1—C6	1.385 (3)
			
Cl1—Zn1—Cl1^i^	110.82 (2)	C4—C5—C6	120.8 (2)
N1—Zn1—N2	85.53 (8)	C6—C5—H5	119.6
N1—Zn1—Cl1	113.82 (3)	C4—C5—H5	119.6
N2—Zn1—Cl1	115.42 (3)	C5—C4—C3	119.7 (2)
N2—Zn1—Cl1^i^	115.42 (3)	C5—C4—H4	120.2
N1—Zn1—Cl1^i^	113.82 (3)	C3—C4—H4	120.2
Zn1—N2—H2	110.1 (15)	C2—C3—C4	120.0 (2)
C2—N2—H2	111.3 (15)	C4—C3—H3	120.0
C2—N2—Zn1	108.56 (13)	C2—C3—H3	120.0
Zn1—N1—H1	111.1 (14)	C3—C2—C1	119.8 (2)
C1—N1—H1	107.8 (14)	C3—C2—N2	121.4 (2)
C1—N1—Zn1	108.67 (13)	C1—C2—N2	118.81 (18)
C5—C6—C1	119.9 (2)	C6—C1—C2	119.8 (2)
C5—C6—H6	120.1	C6—C1—N1	121.79 (19)
C1—C6—H6	120.1	C2—C1—N1	118.43 (19)

Symmetry code: (i) *x*, −*y*+3/2, *z*.

### (Ia) (Benzene-1,2-diamine-κ2N,N')dichloroidozinc Hydrogen-bond geometry (Å, º)

**Table:**

*D*—H···*A*	*D*—H	H···*A*	*D*···*A*	*D*—H···*A*
N1—H1···Cl1^ii^	0.86 (2)	2.59 (2)	3.3618 (16)	150.7 (18)
N2—H2···Cl1^iii^	0.85 (2)	2.52 (2)	3.3204 (16)	157 (2)

Symmetry codes: (ii) *x*+1/2, *y*, −*z*+1/2; (iii) −*x*, −*y*+1, −*z*+1.

### (II) trans-Diaquabis(4,5-dimethylbenzene-1,2-diamine-κ2N,N')zinc chloride–4,5-dimethylbenzene-1,2-diamine (1/2) Crystal data

**Table:**

[Zn(C_8_H_12_N_2_)_2_(H_2_O)_2_]Cl_2_·2C_8_H_12_N_2_	*F*(000) = 760
*M**_r_* = 717.08	*D*_x_ = 1.298 Mg m^−^^3^
Monoclinic, *P*2_1_/*c*	Mo *K*α radiation, λ = 0.71073 Å
*a* = 18.529 (2) Å	Cell parameters from 8719 reflections
*b* = 12.6227 (16) Å	θ = 2.7–25.8°
*c* = 7.8691 (8) Å	µ = 0.86 mm^−^^1^
β = 94.665 (4)°	*T* = 200 K
*V* = 1834.4 (4) Å^3^	Plate, clear colourless
*Z* = 2	0.60 × 0.40 × 0.10 mm

### (II) trans-Diaquabis(4,5-dimethylbenzene-1,2-diamine-κ2N,N')zinc chloride–4,5-dimethylbenzene-1,2-diamine (1/2) Data collection

**Table:**

Bruker SMART X2S benchtop diffractometer	3619 independent reflections
Radiation source: sealed microfocus tube	2920 reflections with *I* > 2σ(*I*)
Doubly curved silicon crystal monochromator	*R*_int_ = 0.060
Detector resolution: 8.3330 pixels mm^-1^	θ_max_ = 26.0°, θ_min_ = 2.7°
/w scans	*h* = −22→22
Absorption correction: multi-scan (SADABS; Bruker, 2013)	*k* = −15→15
*T*_min_ = 0.66, *T*_max_ = 0.92	*l* = −9→9
25430 measured reflections	

### (II) trans-Diaquabis(4,5-dimethylbenzene-1,2-diamine-κ2N,N')zinc chloride–4,5-dimethylbenzene-1,2-diamine (1/2) Refinement

**Table:**

Refinement on *F*^2^	0 restraints
Least-squares matrix: full	Hydrogen site location: mixed
*R*[*F*^2^ > 2σ(*F*^2^)] = 0.031	H atoms treated by a mixture of independent and constrained refinement
*wR*(*F*^2^) = 0.079	*w* = 1/[σ^2^(*F*_o_^2^) + (0.0325*P*)^2^ + 0.4174*P*] where *P* = (*F*_o_^2^ + 2*F*_c_^2^)/3
*S* = 1.05	(Δ/σ)_max_ = 0.001
3619 reflections	Δρ_max_ = 0.31 e Å^−^^3^
245 parameters	Δρ_min_ = −0.22 e Å^−^^3^

### (II) trans-Diaquabis(4,5-dimethylbenzene-1,2-diamine-κ2N,N')zinc chloride–4,5-dimethylbenzene-1,2-diamine (1/2) Special details

**Table:**

Geometry. All esds (except the esd in the dihedral angle between two l.s. planes) are estimated using the full covariance matrix. The cell esds are taken into account individually in the estimation of esds in distances, angles and torsion angles; correlations between esds in cell parameters are only used when they are defined by crystal symmetry. An approximate (isotropic) treatment of cell esds is used for estimating esds involving l.s. planes.

### (II) trans-Diaquabis(4,5-dimethylbenzene-1,2-diamine-κ2N,N')zinc chloride–4,5-dimethylbenzene-1,2-diamine (1/2) Fractional atomic coordinates and isotropic or equivalent isotropic displacement parameters (Å^2^)

**Table:**

	*x*	*y*	*z*	*U*_iso_*/*U*_eq_	
Zn1	0.5	0.5	0.5	0.02953 (11)	
Cl1	0.45668 (3)	0.33892 (4)	1.02374 (6)	0.03564 (13)	
O1	0.56720 (9)	0.42255 (13)	0.31090 (19)	0.0392 (4)	
H1WA	0.6043 (14)	0.447 (2)	0.278 (3)	0.051 (8)*	
H1WB	0.5410 (14)	0.403 (2)	0.231 (3)	0.058 (8)*	
N1	0.40508 (8)	0.41964 (13)	0.4016 (2)	0.0274 (3)	
H1A	0.4163 (11)	0.3837 (17)	0.317 (3)	0.033*	
H1B	0.3870 (11)	0.3749 (17)	0.468 (3)	0.033*	
N2	0.45593 (9)	0.61470 (14)	0.3192 (2)	0.0322 (4)	
H2A	0.4637 (11)	0.6792 (18)	0.355 (3)	0.039*	
H2B	0.4755 (12)	0.6043 (17)	0.233 (3)	0.039*	
N3	0.67491 (11)	0.80052 (17)	0.8184 (3)	0.0422 (4)	
H3A	0.6415 (14)	0.807 (2)	0.741 (3)	0.056 (8)*	
H3B	0.6781 (12)	0.736 (2)	0.859 (3)	0.050 (7)*	
N4	0.68407 (11)	0.98800 (18)	0.6276 (3)	0.0408 (4)	
H4A	0.6917 (13)	1.035 (2)	0.560 (3)	0.058 (8)*	
H4B	0.6509 (14)	0.947 (2)	0.579 (3)	0.053 (7)*	
C1	0.35297 (10)	0.49852 (14)	0.3405 (2)	0.0261 (4)	
C2	0.37896 (10)	0.59711 (15)	0.2950 (2)	0.0280 (4)	
C3	0.33004 (11)	0.67499 (16)	0.2373 (2)	0.0356 (5)	
H3	0.3479	0.7416	0.2031	0.043*	
C4	0.25549 (11)	0.65820 (17)	0.2281 (2)	0.0385 (5)	
C5	0.22942 (10)	0.56000 (18)	0.2798 (2)	0.0373 (5)	
C6	0.27863 (10)	0.48108 (16)	0.3337 (2)	0.0325 (5)	
H6	0.2611	0.414	0.3666	0.039*	
C7	0.20454 (14)	0.7463 (2)	0.1635 (3)	0.0584 (7)	
H7A	0.2328	0.8085	0.1351	0.088*	
H7B	0.1726	0.7651	0.2522	0.088*	
H7C	0.1753	0.7221	0.0615	0.088*	
C8	0.14910 (12)	0.5376 (2)	0.2831 (3)	0.0575 (7)	
H8A	0.1248	0.5496	0.1694	0.086*	
H8B	0.1285	0.5849	0.3653	0.086*	
H8C	0.1421	0.4638	0.3168	0.086*	
C9	0.74336 (10)	0.84050 (16)	0.7842 (2)	0.0337 (4)	
C10	0.74828 (10)	0.93442 (16)	0.6917 (2)	0.0330 (4)	
C11	0.81603 (12)	0.97694 (17)	0.6739 (3)	0.0389 (5)	
H11	0.8193	1.0404	0.61	0.047*	
C12	0.87968 (11)	0.93093 (19)	0.7455 (3)	0.0437 (5)	
C13	0.87500 (12)	0.83640 (19)	0.8363 (3)	0.0441 (5)	
C14	0.80689 (12)	0.79322 (18)	0.8537 (2)	0.0403 (5)	
H14	0.8036	0.7289	0.9154	0.048*	
C15	0.95146 (14)	0.9844 (2)	0.7237 (4)	0.0706 (8)	
H15A	0.9767	0.9977	0.836	0.106*	
H15B	0.9429	1.0518	0.6636	0.106*	
H15C	0.9812	0.9384	0.6575	0.106*	
C16	0.94172 (14)	0.7809 (3)	0.9159 (3)	0.0710 (8)	
H16A	0.9745	0.7651	0.8275	0.107*	
H16B	0.9275	0.7147	0.9691	0.107*	
H16C	0.9664	0.8268	1.0026	0.107*	

### (II) trans-Diaquabis(4,5-dimethylbenzene-1,2-diamine-κ2N,N')zinc chloride–4,5-dimethylbenzene-1,2-diamine (1/2) Atomic displacement parameters (Å^2^)

**Table:**

	*U*^11^	*U*^22^	*U*^33^	*U*^12^	*U*^13^	*U*^23^
Zn1	0.02417 (16)	0.03331 (19)	0.03039 (18)	−0.00446 (13)	−0.00213 (12)	−0.00003 (13)
Cl1	0.0358 (3)	0.0413 (3)	0.0299 (3)	−0.0041 (2)	0.00360 (19)	0.0004 (2)
O1	0.0308 (8)	0.0510 (10)	0.0362 (9)	−0.0069 (7)	0.0048 (7)	−0.0091 (7)
N1	0.0275 (8)	0.0270 (9)	0.0280 (9)	−0.0030 (7)	0.0028 (7)	−0.0012 (7)
N2	0.0310 (9)	0.0344 (9)	0.0318 (9)	−0.0076 (7)	0.0061 (7)	0.0018 (8)
N3	0.0411 (11)	0.0428 (12)	0.0436 (11)	−0.0114 (9)	0.0094 (9)	−0.0012 (9)
N4	0.0359 (10)	0.0443 (12)	0.0422 (11)	−0.0001 (9)	0.0027 (8)	0.0053 (10)
C1	0.0261 (9)	0.0311 (10)	0.0209 (9)	−0.0002 (8)	0.0004 (7)	−0.0021 (8)
C2	0.0287 (9)	0.0329 (10)	0.0225 (9)	−0.0024 (8)	0.0019 (7)	0.0002 (8)
C3	0.0435 (11)	0.0314 (11)	0.0318 (11)	0.0007 (9)	0.0024 (9)	0.0035 (8)
C4	0.0401 (11)	0.0471 (13)	0.0279 (10)	0.0131 (10)	0.0005 (8)	−0.0020 (9)
C5	0.0279 (10)	0.0544 (14)	0.0297 (10)	0.0019 (9)	0.0020 (8)	−0.0012 (9)
C6	0.0273 (10)	0.0400 (12)	0.0301 (10)	−0.0055 (8)	0.0024 (8)	0.0004 (8)
C7	0.0582 (15)	0.0603 (17)	0.0559 (15)	0.0239 (13)	−0.0010 (12)	0.0009 (12)
C8	0.0301 (12)	0.0844 (19)	0.0582 (15)	0.0066 (12)	0.0043 (11)	0.0051 (13)
C9	0.0359 (11)	0.0376 (12)	0.0288 (10)	−0.0068 (9)	0.0093 (8)	−0.0070 (9)
C10	0.0354 (11)	0.0364 (11)	0.0276 (10)	−0.0011 (9)	0.0060 (8)	−0.0047 (8)
C11	0.0405 (12)	0.0393 (12)	0.0379 (11)	−0.0076 (9)	0.0089 (9)	0.0030 (9)
C12	0.0342 (11)	0.0583 (15)	0.0394 (12)	−0.0083 (10)	0.0071 (9)	−0.0055 (11)
C13	0.0389 (12)	0.0572 (15)	0.0361 (11)	0.0042 (11)	0.0025 (9)	−0.0024 (10)
C14	0.0493 (13)	0.0380 (12)	0.0342 (11)	0.0031 (10)	0.0069 (9)	0.0025 (9)
C15	0.0383 (14)	0.100 (2)	0.0741 (19)	−0.0179 (14)	0.0070 (13)	0.0068 (16)
C16	0.0503 (15)	0.095 (2)	0.0656 (17)	0.0143 (15)	−0.0073 (13)	0.0084 (16)

### (II) trans-Diaquabis(4,5-dimethylbenzene-1,2-diamine-κ2N,N')zinc chloride–4,5-dimethylbenzene-1,2-diamine (1/2) Geometric parameters (Å, º)

**Table:**

Zn1—N1^i^	2.1214 (15)	C4—C7	1.519 (3)
Zn1—N1	2.1214 (15)	C5—C6	1.393 (3)
Zn1—N2	2.1442 (17)	C5—C8	1.517 (3)
Zn1—N2^i^	2.1442 (17)	C6—H6	0.95
Zn1—O1^i^	2.2409 (15)	C7—H7A	0.98
Zn1—O1	2.2410 (15)	C7—H7B	0.98
O1—H1WA	0.81 (3)	C7—H7C	0.98
O1—H1WB	0.80 (3)	C8—H8A	0.98
N1—C1	1.442 (2)	C8—H8B	0.98
N1—H1A	0.84 (2)	C8—H8C	0.98
N1—H1B	0.85 (2)	C9—C14	1.391 (3)
N2—C2	1.441 (2)	C9—C10	1.398 (3)
N2—H2A	0.87 (2)	C10—C11	1.383 (3)
N2—H2B	0.81 (2)	C11—C12	1.392 (3)
N3—C9	1.411 (3)	C11—H11	0.95
N3—H3A	0.84 (3)	C12—C13	1.397 (3)
N3—H3B	0.87 (3)	C12—C15	1.514 (3)
N4—C10	1.425 (3)	C13—C14	1.392 (3)
N4—H4A	0.81 (3)	C13—C16	1.512 (3)
N4—H4B	0.87 (3)	C14—H14	0.95
C1—C6	1.392 (2)	C15—H15A	0.98
C1—C2	1.392 (3)	C15—H15B	0.98
C2—C3	1.388 (3)	C15—H15C	0.98
C3—C4	1.393 (3)	C16—H16A	0.98
C3—H3	0.95	C16—H16B	0.98
C4—C5	1.403 (3)	C16—H16C	0.98
			
N1^i^—Zn1—N1	180.0	C6—C5—C4	119.21 (18)
N1^i^—Zn1—N2	100.31 (6)	C6—C5—C8	118.7 (2)
N1—Zn1—N2	79.69 (6)	C4—C5—C8	122.1 (2)
N1^i^—Zn1—N2^i^	79.69 (6)	C1—C6—C5	121.32 (19)
N1—Zn1—N2^i^	100.31 (6)	C1—C6—H6	119.3
N2—Zn1—N2^i^	180.0	C5—C6—H6	119.3
N1^i^—Zn1—O1^i^	92.18 (7)	C4—C7—H7A	109.5
N1—Zn1—O1^i^	87.82 (7)	C4—C7—H7B	109.5
N2—Zn1—O1^i^	86.78 (7)	H7A—C7—H7B	109.5
N2^i^—Zn1—O1^i^	93.22 (7)	C4—C7—H7C	109.5
N1^i^—Zn1—O1	87.82 (7)	H7A—C7—H7C	109.5
N1—Zn1—O1	92.18 (7)	H7B—C7—H7C	109.5
N2—Zn1—O1	93.22 (7)	C5—C8—H8A	109.5
N2^i^—Zn1—O1	86.78 (7)	C5—C8—H8B	109.5
O1^i^—Zn1—O1	180.00 (5)	H8A—C8—H8B	109.5
Zn1—O1—H1WA	124.6 (18)	C5—C8—H8C	109.5
Zn1—O1—H1WB	108.7 (18)	H8A—C8—H8C	109.5
H1WA—O1—H1WB	110 (2)	H8B—C8—H8C	109.5
C1—N1—Zn1	107.71 (12)	C14—C9—C10	118.65 (18)
C1—N1—H1A	108.0 (14)	C14—C9—N3	121.1 (2)
Zn1—N1—H1A	107.1 (14)	C10—C9—N3	120.01 (19)
C1—N1—H1B	111.9 (14)	C11—C10—C9	118.68 (19)
Zn1—N1—H1B	116.6 (13)	C11—C10—N4	121.26 (19)
H1A—N1—H1B	105 (2)	C9—C10—N4	119.93 (18)
C2—N2—Zn1	107.66 (11)	C10—C11—C12	122.9 (2)
C2—N2—H2A	108.9 (14)	C10—C11—H11	118.5
Zn1—N2—H2A	111.8 (14)	C12—C11—H11	118.5
C2—N2—H2B	111.9 (15)	C11—C12—C13	118.52 (19)
Zn1—N2—H2B	106.0 (16)	C11—C12—C15	119.4 (2)
H2A—N2—H2B	111 (2)	C13—C12—C15	122.0 (2)
C9—N3—H3A	116.8 (17)	C14—C13—C12	118.65 (19)
C9—N3—H3B	111.6 (16)	C14—C13—C16	119.7 (2)
H3A—N3—H3B	112 (2)	C12—C13—C16	121.7 (2)
C10—N4—H4A	113.0 (18)	C9—C14—C13	122.6 (2)
C10—N4—H4B	114.7 (17)	C9—C14—H14	118.7
H4A—N4—H4B	107 (2)	C13—C14—H14	118.7
C6—C1—C2	119.58 (17)	C12—C15—H15A	109.5
C6—C1—N1	122.50 (17)	C12—C15—H15B	109.5
C2—C1—N1	117.84 (16)	H15A—C15—H15B	109.5
C3—C2—C1	119.14 (17)	C12—C15—H15C	109.5
C3—C2—N2	123.19 (18)	H15A—C15—H15C	109.5
C1—C2—N2	117.59 (16)	H15B—C15—H15C	109.5
C2—C3—C4	121.84 (19)	C13—C16—H16A	109.5
C2—C3—H3	119.1	C13—C16—H16B	109.5
C4—C3—H3	119.1	H16A—C16—H16B	109.5
C3—C4—C5	118.86 (18)	C13—C16—H16C	109.5
C3—C4—C7	119.5 (2)	H16A—C16—H16C	109.5
C5—C4—C7	121.6 (2)	H16B—C16—H16C	109.5
			
Zn1—N1—C1—C6	−152.85 (15)	C4—C5—C6—C1	1.3 (3)
Zn1—N1—C1—C2	24.00 (19)	C8—C5—C6—C1	−177.21 (19)
C6—C1—C2—C3	−2.4 (3)	C14—C9—C10—C11	−0.4 (3)
N1—C1—C2—C3	−179.38 (16)	N3—C9—C10—C11	173.98 (18)
C6—C1—C2—N2	174.28 (17)	C14—C9—C10—N4	−176.29 (18)
N1—C1—C2—N2	−2.7 (2)	N3—C9—C10—N4	−1.9 (3)
Zn1—N2—C2—C3	156.68 (15)	C9—C10—C11—C12	−0.7 (3)
Zn1—N2—C2—C1	−19.9 (2)	N4—C10—C11—C12	175.16 (19)
C1—C2—C3—C4	1.8 (3)	C10—C11—C12—C13	1.4 (3)
N2—C2—C3—C4	−174.74 (17)	C10—C11—C12—C15	−178.3 (2)
C2—C3—C4—C5	0.4 (3)	C11—C12—C13—C14	−1.1 (3)
C2—C3—C4—C7	−179.85 (18)	C15—C12—C13—C14	178.7 (2)
C3—C4—C5—C6	−1.9 (3)	C11—C12—C13—C16	179.2 (2)
C7—C4—C5—C6	178.34 (19)	C15—C12—C13—C16	−1.0 (3)
C3—C4—C5—C8	176.51 (19)	C10—C9—C14—C13	0.7 (3)
C7—C4—C5—C8	−3.2 (3)	N3—C9—C14—C13	−173.60 (19)
C2—C1—C6—C5	0.9 (3)	C12—C13—C14—C9	0.0 (3)
N1—C1—C6—C5	177.72 (17)	C16—C13—C14—C9	179.7 (2)

Symmetry code: (i) −*x*+1, −*y*+1, −*z*+1.

### (II) trans-Diaquabis(4,5-dimethylbenzene-1,2-diamine-κ2N,N')zinc chloride–4,5-dimethylbenzene-1,2-diamine (1/2) Hydrogen-bond geometry (Å, º)

**Table:**

*D*—H···*A*	*D*—H	H···*A*	*D*···*A*	*D*—H···*A*
O1—H1*WA*···N4^ii^	0.81 (3)	2.13 (3)	2.924 (3)	164 (2)
O1—H1*WB*···Cl1^iii^	0.80 (3)	2.31 (3)	3.1083 (17)	173 (2)
N1—H1*A*···Cl1^iii^	0.84 (2)	2.55 (2)	3.3551 (18)	160.3 (18)
N1—H1*B*···N3^iv^	0.85 (2)	2.31 (2)	3.137 (3)	162.6 (18)
N2—H2*A*···Cl1^v^	0.87 (2)	2.63 (2)	3.4401 (19)	155.3 (18)
N2—H2*B*···Cl1^i^	0.81 (2)	2.57 (2)	3.3105 (18)	154 (2)
N3—H3*A*···Cl1^v^	0.84 (3)	2.68 (3)	3.516 (2)	174 (2)
N3—H3*B*···Cl1^vi^	0.87 (3)	2.89 (2)	3.3284 (19)	112.9 (18)
N4—H4*B*···Cl1^v^	0.87 (3)	2.50 (3)	3.355 (2)	171 (2)

Symmetry codes: (i) −*x*+1, −*y*+1, −*z*+1; (ii) *x*, −*y*+3/2, *z*−1/2; (iii) *x*, *y*, *z*−1; (iv) −*x*+1, *y*−1/2, −*z*+3/2; (v) −*x*+1, *y*+1/2, −*z*+3/2; (vi) −*x*+1, −*y*+1, −*z*+2.

### (Ib) (Benzene-1,2-diamine-κ2N,N')dichloroidozinc Crystal data

**Table:**

[ZnCl_2_(C_6_H_8_N_2_)]	*D*_x_ = 1.808 Mg m^−^^3^
*M**_r_* = 244.41	Mo *K*α radiation, λ = 0.71073 Å
Orthorhombic, *P**n**m**a*	Cell parameters from 2561 reflections
*a* = 8.4152 (12) Å	θ = 2.8–29.2°
*b* = 7.5141 (9) Å	µ = 3.27 mm^−^^1^
*c* = 14.199 (2) Å	*T* = 200 K
*V* = 897.8 (2) Å^3^	Prism, clear colourless
*Z* = 4	0.60 × 0.30 × 0.20 mm
*F*(000) = 488	

### (Ib) (Benzene-1,2-diamine-κ2N,N')dichloroidozinc Data collection

**Table:**

Bruker SMART X2S benchtop diffractometer	1090 independent reflections
Radiation source: XOS X-beam microfocus source	992 reflections with *I* > 2σ(*I*)
Doubly curved silicon crystal monochromator	*R*_int_ = 0.040
Detector resolution: 8.3330 pixels mm^-1^	θ_max_ = 27.5°, θ_min_ = 2.8°
ω scans	*h* = −10→5
Absorption correction: multi-scan (SADABS; Bruker, 2013)	*k* = −8→9
*T*_min_ = 0.40, *T*_max_ = 0.56	*l* = −15→18
4392 measured reflections	

### (Ib) (Benzene-1,2-diamine-κ2N,N')dichloroidozinc Refinement

**Table:**

Refinement on *F*^2^	Primary atom site location: structure-invariant direct methods
Least-squares matrix: full	Secondary atom site location: difference Fourier map
*R*[*F*^2^ > 2σ(*F*^2^)] = 0.027	Hydrogen site location: mixed
*wR*(*F*^2^) = 0.074	H atoms treated by a mixture of independent and constrained refinement
*S* = 1.16	*w* = 1/[σ^2^(*F*_o_^2^) + (0.0377*P*)^2^] where *P* = (*F*_o_^2^ + 2*F*_c_^2^)/3
1090 reflections	(Δ/σ)_max_ < 0.001
72 parameters	Δρ_max_ = 0.41 e Å^−^^3^
0 restraints	Δρ_min_ = −0.58 e Å^−^^3^

### (Ib) (Benzene-1,2-diamine-κ2N,N')dichloroidozinc Special details

**Table:**

Geometry. All esds (except the esd in the dihedral angle between two l.s. planes) are estimated using the full covariance matrix. The cell esds are taken into account individually in the estimation of esds in distances, angles and torsion angles; correlations between esds in cell parameters are only used when they are defined by crystal symmetry. An approximate (isotropic) treatment of cell esds is used for estimating esds involving l.s. planes.
Refinement. Refined as a 2-component inversion twin.

### (Ib) (Benzene-1,2-diamine-κ2N,N')dichloroidozinc Fractional atomic coordinates and isotropic or equivalent isotropic displacement parameters (Å^2^)

**Table:**

	*x*	*y*	*z*	*U*_iso_*/*U*_eq_	
Zn1	0.12938 (3)	0.75	0.38034 (2)	0.03201 (15)	
N1	0.3669 (2)	0.75	0.3494 (2)	0.0343 (6)	
H1	0.388 (2)	0.661 (3)	0.318 (2)	0.040 (7)*	
N2	0.2002 (3)	0.75	0.51812 (18)	0.0354 (6)	
H2	0.165 (3)	0.666 (3)	0.550 (2)	0.047 (7)*	
Cl1	0.00184 (5)	0.50585 (6)	0.33279 (4)	0.04089 (18)	
C1	0.4559 (3)	0.75	0.4370 (2)	0.0294 (6)	
C2	0.3728 (3)	0.75	0.5215 (2)	0.0284 (6)	
C3	0.4543 (4)	0.75	0.6066 (2)	0.0402 (7)	
H3	0.3973	0.75	0.6644	0.048*	
C4	0.6198 (4)	0.75	0.6068 (3)	0.0482 (9)	
H4	0.6761	0.75	0.6648	0.058*	
C5	0.7015 (4)	0.75	0.5231 (3)	0.0493 (8)	
H5	0.8144	0.75	0.5237	0.059*	
C6	0.6220 (3)	0.75	0.4382 (3)	0.0423 (8)	
H6	0.6798	0.75	0.3807	0.051*	

### (Ib) (Benzene-1,2-diamine-κ2N,N')dichloroidozinc Atomic displacement parameters (Å^2^)

**Table:**

	*U*^11^	*U*^22^	*U*^33^	*U*^12^	*U*^13^	*U*^23^
Zn1	0.0301 (2)	0.0408 (3)	0.0251 (2)	0	−0.00244 (10)	0
N1	0.0345 (12)	0.0468 (18)	0.0216 (12)	0	0.0024 (8)	0
N2	0.0346 (12)	0.0469 (17)	0.0246 (12)	0	0.0041 (9)	0
Cl1	0.0502 (3)	0.0367 (3)	0.0358 (3)	−0.0066 (2)	−0.0058 (2)	0.0032 (2)
C1	0.0341 (12)	0.0282 (14)	0.0259 (13)	0	−0.0001 (10)	0
C2	0.0309 (13)	0.0289 (14)	0.0253 (14)	0	−0.0005 (9)	0
C3	0.0498 (16)	0.0423 (17)	0.0283 (15)	0	−0.0035 (12)	0
C4	0.0487 (18)	0.051 (2)	0.045 (2)	0	−0.0201 (14)	0
C5	0.0331 (14)	0.053 (2)	0.061 (2)	0	−0.0120 (14)	0
C6	0.0331 (14)	0.051 (2)	0.0425 (19)	0	0.0048 (11)	0

### (Ib) (Benzene-1,2-diamine-κ2N,N')dichloroidozinc Geometric parameters (Å, º)

**Table:**

Zn1—Cl1	2.2301 (5)	C5—C6	1.379 (5)
Zn1—Cl1^i^	2.2301 (5)	C5—H5	0.95
Zn1—N1	2.047 (2)	C4—C5	1.373 (5)
Zn1—N2	2.045 (3)	C4—H4	0.95
N2—C2	1.453 (3)	C3—C4	1.393 (4)
N2—H2	0.83 (3)	C3—H3	0.95
N1—C1	1.452 (4)	C2—C3	1.389 (4)
N1—H1	0.83 (3)	C1—C2	1.388 (4)
C6—H6	0.95	C1—C6	1.398 (3)
			
Cl1—Zn1—Cl1^i^	110.70 (3)	C4—C5—C6	120.9 (3)
N1—Zn1—N2	85.45 (10)	C6—C5—H5	119.5
N1—Zn1—Cl1	113.89 (4)	C4—C5—H5	119.5
N2—Zn1—Cl1^i^	115.46 (3)	C5—C4—C3	119.9 (3)
N2—Zn1—Cl1	115.46 (3)	C5—C4—H4	120.0
N1—Zn1—Cl1^i^	113.89 (4)	C3—C4—H4	120.0
Zn1—N2—H2	114.1 (19)	C2—C3—C4	119.7 (3)
C2—N2—H2	110.0 (17)	C4—C3—H3	120.2
C2—N2—Zn1	108.84 (19)	C2—C3—H3	120.2
Zn1—N1—H1	109.0 (14)	C1—C2—C3	120.2 (2)
C1—N1—H1	111.0 (18)	C3—C2—N2	121.5 (3)
C1—N1—Zn1	108.62 (18)	C1—C2—N2	118.4 (2)
C5—C6—C1	119.7 (3)	C2—C1—C6	119.6 (3)
C5—C6—H6	120.2	C6—C1—N1	121.7 (3)
C1—C6—H6	120.2	C2—C1—N1	118.7 (2)
			
Zn1—N1—C1—C2	0	C1—C2—C3—C4	0.0000 (10)
Zn1—N1—C1—C6	180.0	N2—C2—C3—C4	180.0000 (10)
C6—C1—C2—C3	0.0000 (10)	C2—C3—C4—C5	0.0000 (10)
N1—C1—C2—C3	180.0000 (10)	C3—C4—C5—C6	0.0000 (10)
C6—C1—C2—N2	180.0	C4—C5—C6—C1	0.0000 (10)
N1—C1—C2—N2	0.0000 (10)	C2—C1—C6—C5	0.0000 (10)
Zn1—N2—C2—C1	0.0000 (10)	N1—C1—C6—C5	180.0000 (10)
Zn1—N2—C2—C3	180.0000 (10)		

Symmetry code: (i) *x*, −*y*+3/2, *z*.

### (Ib) (Benzene-1,2-diamine-κ2N,N')dichloroidozinc Hydrogen-bond geometry (Å, º)

**Table:**

*D*—H···*A*	*D*—H	H···*A*	*D*···*A*	*D*—H···*A*
N1—H1···Cl1^ii^	0.83 (3)	2.61 (3)	3.368 (2)	152 (2)
N2—H2···Cl1^iii^	0.83 (3)	2.53 (3)	3.327 (2)	160 (2)

Symmetry codes: (ii) *x*+1/2, *y*, −*z*+1/2; (iii) −*x*, −*y*+1, −*z*+1.
